# Prognostic value of increased expression of RBM8A in gastric cancer

**DOI:** 10.1590/1414-431X20209290

**Published:** 2020-04-09

**Authors:** Xinting Lv, Huifei Cheng

**Affiliations:** 1 Department of Surgery, The First People's Hospital of Yongkang, Zhejiang, China; 2 Department of Radiotherapy, Lishui Municipal Central Hospital, Zhejiang, China

**Keywords:** Gastric cancer, RBM8A, Proto-oncogene, Prognosis

## Abstract

This study was designed to investigate the expression of RBM8A protein in patients with gastric cancer (GC) and to explore its correlation with clinical pathological features as well as prognosis. One hundred pairs of gastric carcinoma tissues and adjacent tissues from patients undergoing gastrectomy for GC were included in this study. The protein expression level of RBM8A was determined by immunohistochemistry using tissue microarrays. We also detected the mRNA expression level of RBM8A in 16 pairs of gastric carcinoma tissues and adjacent tissues. Meanwhile, we predicted the potential correlation between RBM8A and tumor stages as well as survival condition in patents with GC based on The Cancer Genome Atlas (TCGA) database. The correlation of RBM8A with the clinical pathological features and prognosis of the 100 patients with GC was also elucidated. The expression level of RBM8A was significantly higher in gastric carcinoma tissues compared to the adjacent tissues. The protein level of RBM8A was correlated with tumor size (P=0.031), depth of invasion (P<0.001), lymph node metastasis (P<0.001), TNM stage (<0.001), and distant metastasis (P=0.001). Patients with increased RBM8A expression (P<0.0018, 95%CI=0.322−0.871), higher TNM stage (P<0.001, 95%CI=4.990−11.283), and lymph node metastasis (P<0.001, 95%CI=2.873−4.002) had a lower overall survival. Taken together, our study demonstrated that RBM8A may act as a proto-oncogene, which could be a promising biomarker and therapeutic target in the diagnosis and treatment of GC.

## Introduction

Gastric cancer (GC) is still the second leading cause of cancer-related deaths globally although the incidence of GC has been decreasing over the past decades (1). In particular, GC cases occurring in China and other developing countries account for 70% of global cases (2). Early diagnosis and surgical resection of carcinoma tissue confined to the stomach provides the best hope for cure or extension of lifespan of patients with GC (3). Although great breakthroughs in diagnosis and treatment of GC have been achieved, the 5-year survival of patients with GC still remains high (4). Additionally, the efficiency and accuracy in screening patients with asymptomatic diseases are unsatisfactory (5). Hence, an integrated understanding of molecular mechanisms underlying GC may improve prognosis, and also assist to uncover more practical prognostic biomarkers and identify potent chemotherapeutic targets.

RNA binding motif protein 8A (RBM8A, also known as Y14), one of the members in the RNA-binding motif protein (RBM) family, contains an RNA-recognition motif and heterodimerizes with Mago to form the exon junction complex (EJC) assembling spliced mRNAs (6,7). Previous studies demonstrated that RBM8A is involved in cell differentiation, apoptosis, RNA splicing, cycle regulation, and played a vital role in the development and physiology of mammals. Additionally, RBM8A can govern proto-oncogene p53 expression and modify DNA damage sensitivity, thereby affecting the development of tumors. Loss of RBM8A suppressed the proliferation of A549 cells and Hela cells by restricting them at the M phase (8). Meanwhile, abnormal apoptotic centrosomes were also found in cancer cells after the expression of RBM8A was inhibited (9). Although the expression of RBM8A in other cancers has been investigated, the expression and biologic roles of RBM8A in GC have not been reported.

In the present study, we sought to detect the vital expression of RBM8A protein in gastric carcinoma tissues and adjacent specimens, and further analyze its expression level with the clinicopathological features and prognosis. RBM8A expression was measured using immunohistochemistry and qRT-PCR techniques. We also used the public TCGA (The Cancer Genome Atlas) database to study the expression level of RBM8A in GC, and analyzed the relationship between its expression and tumor stages, as well as prognosis.

## Material and Methods

### Patient data

A total of 100 surgical tissue samples of GC were collected from patients at the First People's Hospital of Yongkang between January 2014 and January 2018 after the subjects’ informed consent and institutional review board approval were acquired. All enrolled patients were previously diagnosed by histopathological analysis by 2 senior pathologists. Clinical data of sex, age, histological type, differentiation grade, and TNM stage were recorded. The patient cohort consisted of 62 males and 38 females, with a median age of 66.8±4.82 years (range: 32–94 years). Among these 100 GC cases, 46 were from the gastric body, 24 from the cardia, and 30 from the antrum. Based on the World Health Organization (WHO) histological classification of GC (2), 44 cases were identified as tubular, 26 cases as papillary, 20 cases as signet-ring cell, and 10 cases as mucinous adenocarcinomas. According to the Lauren classification of GC, 55 cases were diffuse-type, 33 intestinal-type, and 12 mixed type. As described in the 7th edition of the Union for International Cancer Control Tumor-Node-Metastasis (TNM) classification system for GC, 11 cases were in stage I, 20 cases stage II, and 69 cases stage III. One hundred pairs of CG tissues and adjacent tissues were formalin-fixed paraffin-embedded (FFPE) and carcinoma tissues were further processed for tissue microarray (TMA).

### TMA construction and immunohistochemistry

A total 8 gastric TMAs (Shanghai Outdo Biotech, China) were used in this study. Core tissue biopsies (2 mm in diameter) were acquired from ∼100 individual FFPE blocks. Subsequently, these FFPE blocks were arranged in one prepared blank paraffin block. After that, 4-micron thick sections were cut and placed on super frost-charged glass microscope slides to obtain TMA slides. Immunohistochemical staining was carried out on these TMA slides as previously reported (10). In detail, the tissue sections were deparaffinized and rehydrated with graded alcohol. Subsequently, the tissue sections were incubated with 10% normal goat serum (Beijing Solarbio Science & Technology Co., Ltd., China) for 10 min at room temperature to decrease nonspecific reactions. Then, the tissue sections were incubated with the primary antibody to RBM8A (1:200 dilution; Abcam, UK) overnight at 4°C. The next day, the sections were washed 3 times with PBS. Then, the TMA sections were incubated with secondary antibody (LI-COR Biosciences, USA) at room temperature for 20 min and stained with diaminobenzidine (DAB)-H_2_O_2_. The TMA sections were then counterstained with hematoxylin and mounted on a coverslip. Finally, the sections were viewed under an optical microscope (Nikon H550L microscope, Japan).

RBM8A protein expression was examined by the proportion of immunoreactive cells and the staining intensity with the semi-quantitative H-score method as follows: the proportion of immunoreactive cells was scored as 3 (≥51% positive cells), 2 (26–50% positive cells), 1 (6–25% positive cells), and 0 (≤5% positive cells), and the intensity of immunostaining was scored as 3 (intense staining, brown), 2 (moderate staining, yellowish brown), 1 (weak staining, light yellow), and 0 (no staining). Immunohistochemical staining was examined independently by 2 experienced pathologists who were blinded to experimental conditions.

### Real-time PCR

Total RNA was extracted from gastric tissues using Trizol (Invitrogen, USA) based on the manufacturer's protocol (11). Subsequently, the cDNA was amplified by a reverse transcriptional kit (Promega, USA). The real-time PCR was performed using cDNA as a template and Universal PCR Master Mix (Applied Biosystems, USA) by an Applied Biosystems 7900HT sequence detection system. The relative amount of mRNA was calculated and normalized using GAPDH as internal reference.

### Gene Expression Profiling Interactive Analysis (GEPIA) dataset

GEPIA (http://gepia.cancer-pku.cn/index.html) serves as a newly generated interactive web server designed by Zefang Tang, Chenwei Li, and Boxi Kang of Zhang Lab, Peking University to analyze the RNA sequencing expression data of 9736 tumors and 8587 normal samples from the GTEx projects and TCGA database in a standard-processing manner. GEPIA provides customizable functions including tumor/normal differential expression analysis, profiling according to cancer types or pathological stages, patient survival analysis, similar gene detection, correlation analysis, and dimensionality reduction analysis (12). In this study, we mainly employed the boxplot to detect the mRNA expression of RBM8A genes in GC and normal gastric tissues. Additionally, the stage plot and survival condition of patients with different levels of RBM8A were obtained. The log-rank P value and hazard ratio (HR) with 95% confidence intervals are shown on the plot. P<0.05 was statistically significant.

### Human Protein Atlas

The Human Protein Atlas (HPA, https://www.proteinatlas.org/) is a Swedish-based program initiated in 2003 for mapping all human proteins in cells, tissues, and organs using the integration of various omics technologies, including antibody-based imaging, mass spectrometry-based proteomics, transcriptomics, and systems biology (13). By getting immunohistochemical data of patients with or without GC on the basis of HPA, we further verified the protein expression levels of RBM8A.

### Statistical analysis

The obtained quantitative data are reported as means±SD and assessed by the two-tailed Student's *t*-test. Categorical data were analyzed using the chi-squared test or Fisher's exact test. A difference of P<0.05 was considered statistically significant. Multivariate survival analysis was performed using the Cox proportional hazards model, and variables that were significant in the univariate analysis were included in the model with the Enter method.

## Results

### Increased RBM8A mRNA expression in GC

**Figure 1:**
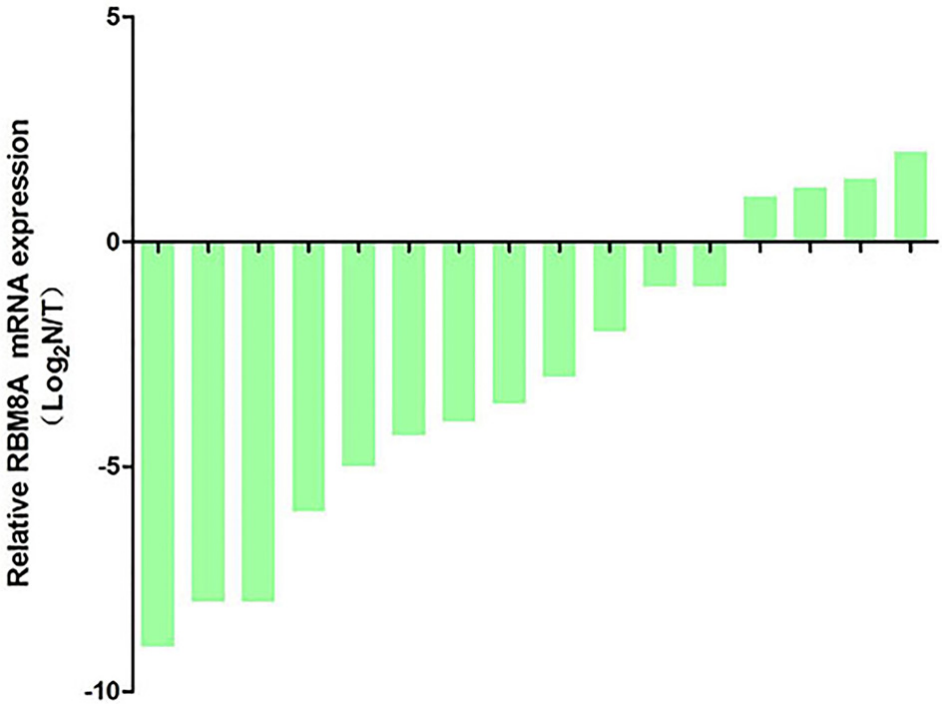
mRNA expression of RBM8A in 16 paired gastric carcinoma samples and adjacent tissues. Data are reported as the ratio of adjacent tissues (N)/carcinoma samples (T).

We detected RBM8A mRNA levels in 16 paired adjacent tissues and gastric carcinoma tissues. The results from real-time PCR demonstrated that 12 paired gastric cancer samples had significantly increased RBM8A expression levels compared to the paired adjacent tissues at mRNA levels, with an average upregulation fold of 6.89 (P<0.001) (Figure 1).

### TPM and transcriptional levels of RBM8A gene in patients with GC based on TCGA database

**Figure 2:**
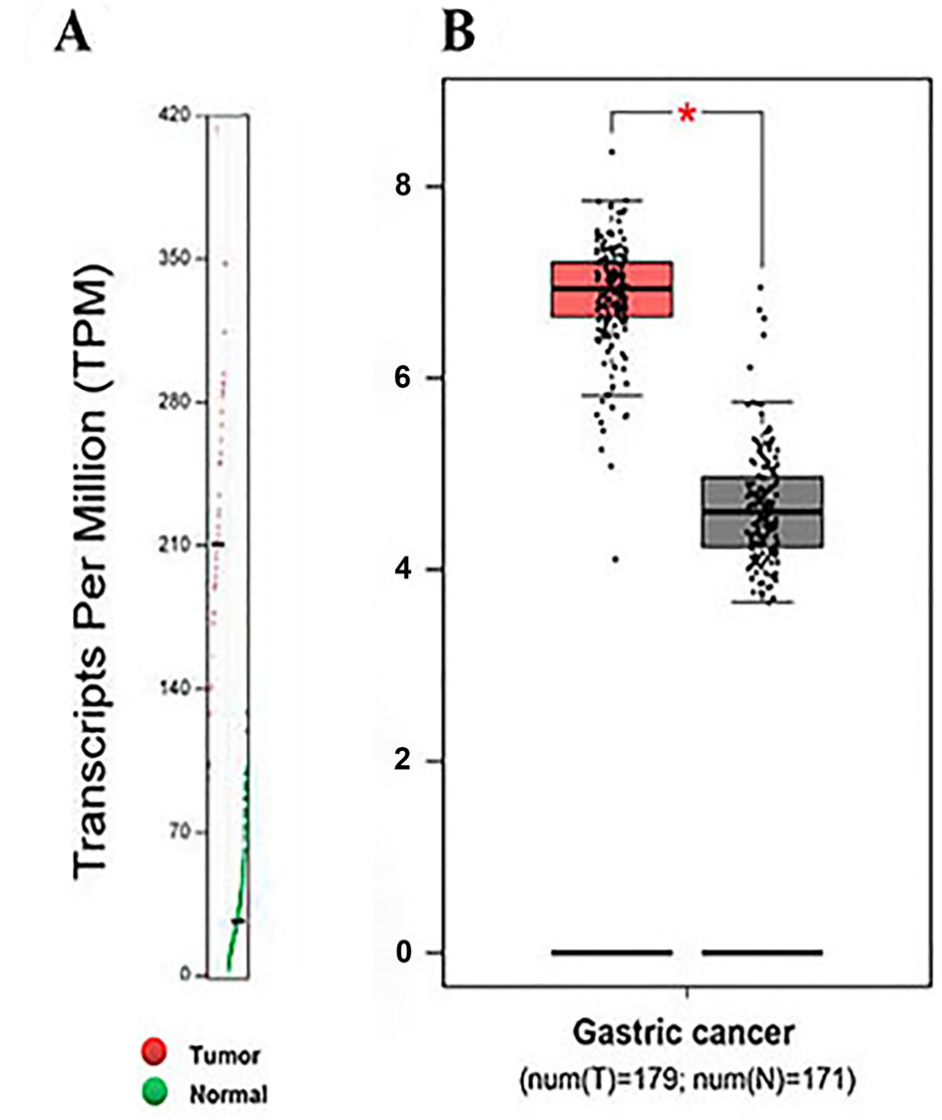
Transcripts per million (TPM) and mRNA levels of RBM8A in patients with gastric cancer and normal controls, *P<0.05 *vs* Normal group. Data were analyzed by the two-tailed Student's *t*-test.

Transcripts per million (TPM) is a measurement of the proportion of transcripts in the pool of RNA, which is defined as transcripts per kilobase of exon model per million mapped reads. In this study, after analyzing 171 normal gastric tissues and 179 gastric carcinoma tissues based on TCGA database, a higher level of TPM of RBM8A in GC group was observed (P<0.05) (Figure 2A), indicating that RBM8A possessed more transcripts in gastric carcinoma tissues. We also found that the mRNA level of RBM8A was significantly higher in patients with GC than normal control (P<0.05) (Figure 2B).

### Immunohistochemical data of RBM8A based on HPA database

**Figure 3:**
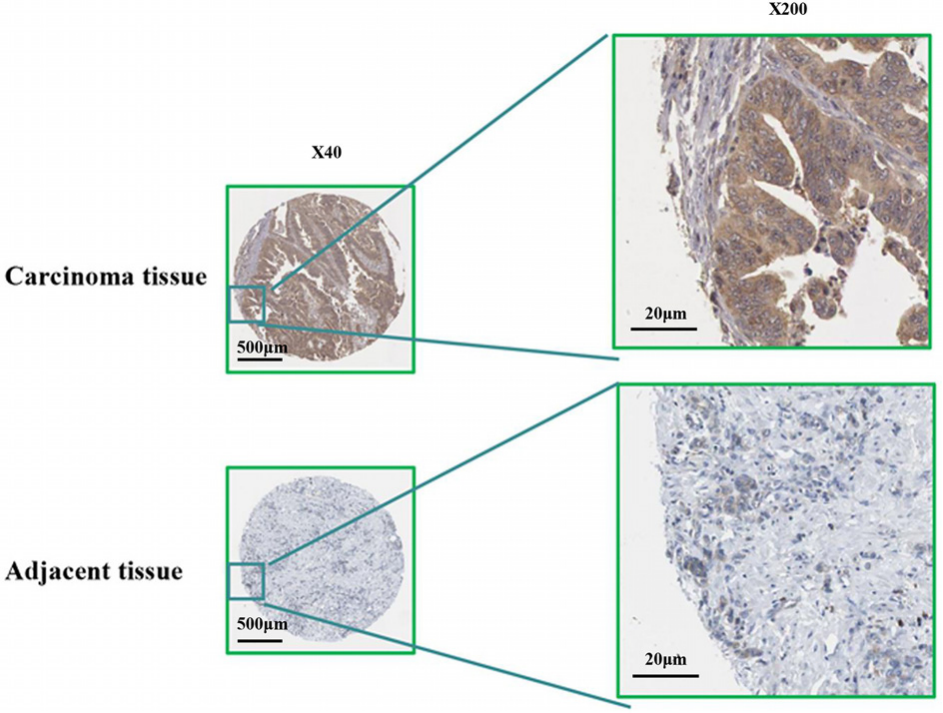
Immunohistochemical staining of RBM8A in gastric carcinoma samples and adjacent tissues. Bars: 500 μm and 20 μm.

To enhance the reliability of the database, we searched the immunohistochemical data in the HPA database. Consistent with the above results, the immunohistochemical data from HPA demonstrated that the protein level of RBM8A was significantly higher in gastric carcinoma tissues compared with adjacent tissue (Figure 3).

### Correlation between RBM8A gene expression and tumor stage in patients with GC based on TCGA database

**Figure 4:**
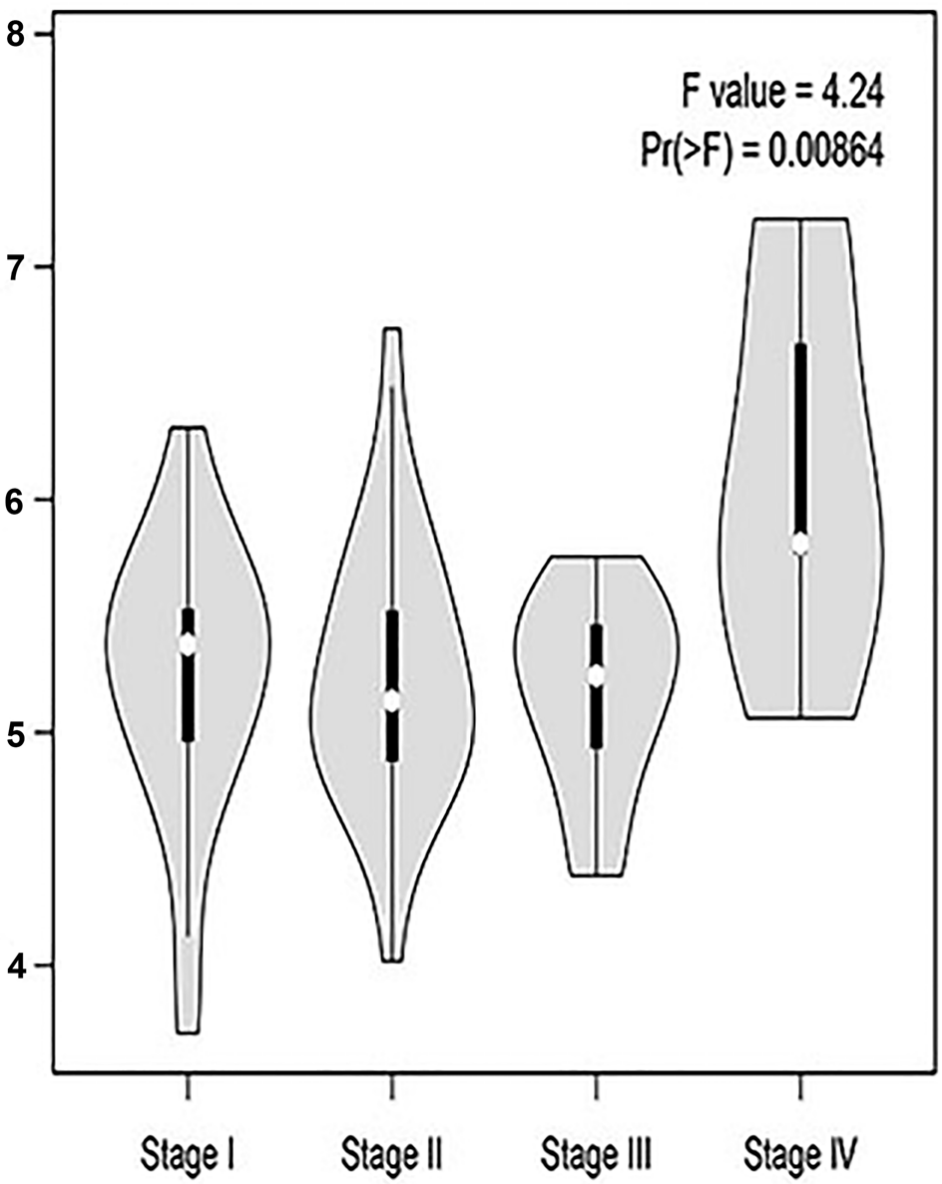
Relationship between the level of RBM8A and tumor stages in patients with gastric cancer.

We analyzed the correlation between RBM8A expression and tumor stage in patients with GC by bioinformatics. The results demonstrated that the expression levels of RBM8A displayed strong correlation with the tumor stages in patients with GC (P<0.05) (Figure 4).

### Correlation between RBM8A gene expression and survival condition in patients with GC based on TCGA database

**Figure 5:**
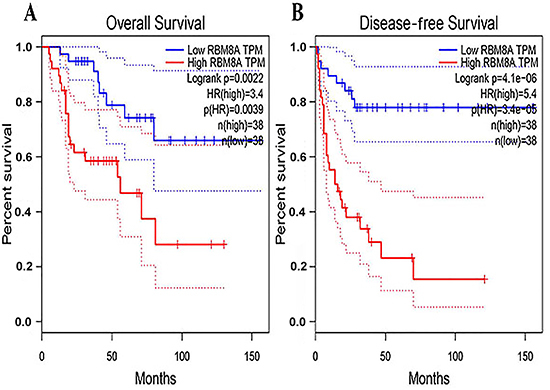
Relationship between the level of RBM8A and overall survival (**A**) and disease-free survival (**B**) in patients with gastric cancer.

We further analyzed the potential association between RBM8A gene expression levels and the survival condition of patients with GC. The Kaplan-Meier test showed that the levels of RBM8A displayed significant correlation with the overall survival and disease-free survival of patients with GC (P<0.05). The lower level of RBM8A contributed to both better overall survival and better disease-free survival of GC patients (Figure 5A and B).

### Correlation of RBM8A protein expression with clinicopathological features

The correlation between RBM8A protein expression and clinicopathological parameters of 100 patients with GC was analyzed. The expression level of RBM8A protein in GC was significantly correlated with tumor size (P=0.031), depth of invasion (P<0.001), lymph node metastasis (P<0.001), TNM stage (P<0.001), and distant metastasis (P=0.001) (Table 1).

**Table 1:** Correlation of RBM8A protein expression level with clinicopathological features in patients with gastric cancer (n=100).

Clinicopathological Data	Total No. patients	RBM38 protein expression level		χ^2^	P value
		Low (n,%)	High (n,%)		
Gender				3.229	0.782
Male	62	34 (54.8%)	28 (45.2%)		
Female	38	16 (42.1%)	22 (57.9%)		
Age (years)				0.331	0.349
≤60	26	18 (69.2%)	8 (30.8%)		
<60	74	38 (51.4%)	36 (48.6%)		
Tumor size				5.192	0.031*
≤5cm	70	32 (45.7%)	38 (54.3%)		
<5cm	30	22 (73.3%)	8 (26.7%)		
Depth of invasion				22.129	<0.001*
T1	15	3 (20%)	12 (80%)		
T2	18	5 (27.8%)	13 (72.2%)		
T3	40	33 (82.5%)	7 (17.5%)		
T4	27	18 (66.7%)	9 (33.3%)		
Lymph node metastasis				18.293	<0.001*
N0	14	2 (14.3%)	12 (85.7%)		
N1	30	11 (36.7%)	19 (63.3%)		
N2	34	30 (88.2%)	4 (11.8%)		
N3	22	20 (90.9%)	2 (9.1%)		
TNM stage				24.932	<0.001*
I	11	3 (27.3%)	8 (72.7%)		
II	20	6 (30%)	14 (70%)		
III	69	50 (72.5%)	19 (27.5%)		
Distant metastasis				12.902	0.001*
M0	88	41 (46.6%)	47 (53.4%)		
M1	12	1 (8.3%)	11 (91.7%)		

TNM: tumor-node-metastasis. *P<0.05 was considered statistically significant (chi-squared test).

### Correlation between RBM8A protein expression level and prognosis of patients with GC

The univariate and multivariate analyses unveiled the prognostic value of RBM8A in patients with GC (Table 2). Increased RBM8A expression (HR=0.421, 95%CI=0.301−0.662; P<0.001) was significantly associated with a poor survival in univariate analysis, along with other prognostic markers, involving age (HR= 2.228, 95%CI=0.522−0.892; P=0.048), tumor size (HR=0.415, 95%CI=0.283−0.781; P< 0.003), TNM stage (HR=7.883, 95%CI=5.839−9.302; P<0.001), lymph node metastasis (HR=1.992, 95%CI=1.672-2.198; P<0.001), and distant metastasis (HR=6.892, 95%CI=2.199−14.212; P<0.001). In multivariate analysis, both increased RBM8A expression (HR=0.470, 95%CI=0.322−0.871; P<0.0018) and lymph node metastasis (HR=3.271, 95%CI=2.873−4.002; P<0.001) induced worse prognosis independently (Table 2).

**Table 2:** Univariate and multivariate analysis of RBM8A for overall survival in patients with gastric cancer.

	Univariate analysis			Multivariate analysis		
	HR	P value	95%CI	HR	P value	95%CI
RBM8A expression	0.421	<0.001*	0.301	0.470	<0.0018	0.322
High *vs* Low or none			0.662			0.871
Age (years)	2.228	0.048*	0.522	0.917	0.562	0.619
≤60 *vs* >60			0.892			1.286
Tumor size	0.415	0.003*	0.283	0.488	0.206	0.569
≤5cm *vs* >5cm			0.781			2.863
Depth of invasion	0.772	0.416	0.116	0.637	0.622	0.412
T1/2 *vs* T3/4			0.629			1.821
TNM stage	7.883	<0.001*	5.839	8.291	<0.001*	4.990
I/II *vs* III/IV			9.302			11.283
Lymph node metastasis	1.992	<0.001*	1.672	3.271	<0.001*	2.873
N0-1 *vs* N2-3			2.198			4.002
Distant metastasis	6.892	<0.001*	2.199	2.119	0.681	0.263
Mo *vs* M1			14.212			1.226

HR: hazard ratio; TNM: tumor-node-metastasis. *P<0.05 was considered statistically significant.

## Discussion

In present study, we found that the RBM8A mRNA and protein expression was increased in gastric carcinoma tissues compared with normal gastric tissues on the basis of immunohistochemistry and real time-PCR analysis. In addition, we determined that high expression levels of RBM8A protein were strongly correlated with worse prognosis of patients with GC. Moreover, we demonstrated that RBM38 may act as a vital proto-oncogene in GC.

RBM8A, as a main encoding RNA binding protein, is located at chromosome 14q21-q23 with a molecular weight of 26 kDa (14,15). The RBM8A gene was found to code 4 transcripts and express widely within various types of cell and could shift between cytoplasm and nucleus (16). Unlike other RNA binding motif proteins, the structure as well as function of RBM8A is incompletely understood. RBM8A is an RNA recognition motif-containing protein that forms heterodimers with MAGOH and serves as a core factor of the RNA surveillance machinery for the exon junction complex. RBM8A is known to be a component of the exon junction complex, which could regulate IL-6-induced STAT3 activation in human cervix carcinoma cell line (17). RBM8A-deficient cells cannot enter the next G1 phase beyond G2/M phase after release from G1/S arrest (9). Also, RBM8A is crucial for proliferation and differentiation of cortical neural progenitor cells by regulating multiple risk genes associated with neurodegenerative or neuropsychiatric diseases (18). Meanwhile, RBM8A was a direct target of miR-29a in retinal progenitors and could regulate its proliferation and differentiation (19).

Additionally, we showed that high expression levels of RBM8A were closely associated with reduced overall survival and disease-free survival in patients with GC. Also, RBM8A was an independent GC prognostic factor according to multivariate Cox regression analysis. Of course, it is not only a single gene affecting each step of the metastasis process in the occurrence and development of GC. Other genes associated with RBM8A in cell and animal models need to be further explored. In hepatocellular carcinoma, the expression level of RBM8A is significantly increased. Moreover, RBM8A exhibited significant differences in tumor diameter, HBsAg expression, Edmondson pathological grading, as well as TNM staging (20).

In summary, we found that RBM8A may act as a potential diagnostic marker and a therapeutic target of GC, which may function as a proto-oncogene. The precise regulatory mechanism of RBM38 in GC needs to be further studied to investigate its potential role and relevance in GC and to implement it as a tumor therapeutic target in GC individual therapy.
